# Geographic Population Structure of the Sugarcane Borer, *Diatraea saccharalis* (F.) (Lepidoptera: Crambidae), in the Southern United States

**DOI:** 10.1371/journal.pone.0110036

**Published:** 2014-10-22

**Authors:** Andrea L. Joyce, William H. White, Gregg S. Nuessly, M. Alma Solis, Sonja J. Scheffer, Matthew L. Lewis, Raul F. Medina

**Affiliations:** 1 SNRI, University of California Merced, Merced, California, United States of America; 2 USDA-ARS Sugarcane Research Unit, Houma, Louisiana, United States of America; 3 University of Florida, Everglades Research and Education Center, Belle Glade, Florida, United States of America; 4 USDA, Systematic Entomology, National Museum of Natural History, Washington, D. C., United States of America; 5 USDA-ARS, Systematic Entomology Lab, Beltsville, Maryland, United States of America; 6 Department of Entomology, Texas A&M University, College Station, Texas, United States of America; Louisiana State University & LSU AgCenter, United States of America

## Abstract

The sugarcane borer moth, *Diatraea saccharalis*, is widespread throughout the Western Hemisphere, and is considered an introduced species in the southern United States. Although this moth has a wide distribution and is a pest of many crop plants including sugarcane, corn, sorghum and rice, it is considered one species. The objective was to investigate whether more than one introduction of *D. saccharalis* had occurred in the southern United States and whether any cryptic species were present. We field collected *D. saccharalis* in Texas, Louisiana and Florida in the southern United States. Two molecular markers, AFLPs and mitochondrial COI, were used to examine genetic variation among these regional populations and to compare the sequences with those available in GenBank and BOLD. We found geographic population structure in the southern United States which suggests two introductions and the presence of a previously unknown cryptic species. Management of *D. saccharalis* would likely benefit from further investigation of population genetics throughout the range of this species.

## Introduction

The sugarcane borer, *Diatraea saccharalis* (Fabricius) (Lepidoptera: Crambidae), is widely distributed in the Western Hemisphere throughout much of South America, Central America, the Caribbean, and the southern United States [1], [2], [3]. The native range of the sugarcane borer is uncertain, as the species has been collected throughout the neotropics on a number of host plants. The wild host plants of *D. saccharalis* are numerous [1], [4] and include some aquatic and riparian species [4], [5]. Cultivated host plants of *D. saccharalis* include crops such as sugarcane (*Saccharum* spp), corn (*Zea mays* L.), sorghum (*Sorghum bicolor* L.) and rice (*Oryza sativa* L.) [6], [7]. In the southern United States, *D. saccharalis* is considered an introduced insect pest [8], [9]. *Diatraea saccharalis* was first reported as a pest in Louisiana around 1854 [10] and was presumed to be introduced from Hispaniola with the introduction of sugarcane [11]. Subsequently, the moth became a pest in Florida in the 1920s and in Texas in 1972 [12], [13], [14], [15]. The damage caused by *D. saccharalis* larvae feeding in its cultivated host plants includes a decrease in plant sugar content and crop yields, reduction of plant biomass, and increased susceptibility to plant pathogens by providing points of pathogen entry [8], [16], [17], [18], [19], [20].

Although *D. saccharalis* has a broad geographic distribution in addition to a wide host plant range [1], [4], it is treated as a single species. Few studies have investigated the existence of cryptic species or the population genetics of this insect [1], [21]. Often species with a widespread distribution warrant further investigation to determine whether they are truly one species [22], or instead consist of a species complex. In addition, this insect may have been moved throughout the Western Hemisphere due to movement of its host plants, and more than one genotype of *D. saccharalis* may have been introduced into the southern United States. Genetically distinct populations of insects can vary in their susceptibility to natural enemies and other control tactics [23], [24]. Determining the population structure of this insect in the southern United States could contribute to its management as well as help identify future introductions and their likely region of origin.

Identification of *Diatraea* species relies on morphological identification as there have been few genetic or molecular studies focusing on *D. saccharalis* [1], [21], [25], [26], [27], [28], [29]. An electrophoretic comparison of enzymes of *D. saccharalis* populations from Louisiana, Mexico, and Brazil found a Nei's genetic distance of 0.23 between the Brazilian population and those from Texas and Louisiana, while the genetic distance between the Mexico and Louisiana populations was only 0.02 [21]. Examination of mitochondrial DNA CO II sequences from *D. saccharalis* populations throughout its range found that a population from Valle del Cauca, Colombia, averaged 2.7% distance from other populations including those from Brazil; however, relationships between other *D. saccharalis* populations were difficult to resolve, perhaps due to the small sample sizes for many populations [29]. A sequence divergence of 2–3% can indicate the presence of another insect species, depending on the insect group in question [30]. Obtaining robust samples sizes of populations of *D. saccharalis* from Central America, the Caribbean and the southern United States could contribute to a more comprehensive analysis of geographic population structure, to explore if *D. saccharalis* consists of a species complex or is indeed one widespread polyphagous species.

In the southern United States, more than one genotype of *D. saccharalis* may have been introduced from different areas of this species range. The insect became a pest in Texas, Louisiana and Florida during different decades over the course of nearly a century. Classical biological control programs which released exotic natural enemies for control of *D. saccharalis* were conducted in the southern US and different parasitoid species established in Texas, Louisiana, and Florida [14], [16], [31], suggesting that the moth genotype could vary among the three regions. The parasitoid fly, *Lixophaga diatraea* Townsend (Diptera: Tachinidae), established in Louisiana but not in Texas or Florida. While the parasitoid wasp, *Cotesia flavipes* Cameron (Hymenoptera: Braconidae) failed to establish in Louisiana, it took hold in Texas and Florida [14], [31], [32]. However, many factors contribute to the establishment of natural enemies introduced for classical biological control, including variation in climate or cultivation practices, as well as genetic variation in populations of the pest or its natural enemies [23], [24], [31].

The objective of this study was to investigate the geographic population structure of *D. saccharalis* in the southern United States, to determine whether these regional populations of *D. saccharalis* are genetically distinct, possibly representing independent introductions and/or cryptic species. We investigated this question by collecting *D. saccharalis* in Texas, Louisiana and Florida and by examining their population structure using amplified fragment length polymorphisms (AFLPs). In addition, a 658 base pair region of the mitochondrial DNA COI gene was sequenced from several individuals from each southern United States population. The mitochondrial COI sequences were compared to publicly available COI sequences for *D. saccharalis*, to investigate potential source populations for those established in the southern US, as well as to estimate the number of potential cryptic species which may exist within this species.

## Methods

### Insect collections

*Diatraea saccharalis* from Texas, Louisiana and Florida were field collected as larvae or adults during 2009–2010. No specific permissions were required for collecting insects in any of these locations, and the field studies did not involve any endangered or protected species. In Louisiana, *D. saccharalis* larvae were collected on sugarcane plants. We first identified sugarcane plants with larval feeding damage (holes in plant stems with larval frass) and then removed larvae from plants, placed them on artificial diet (Southland Products, Lake Village Arkansas) in 60 ml plastic cups, and transported them to the laboratory to rear them into adults. Field collections in Louisiana were made in June and September 2009 at field sites within 200 km of the United States Department of Agriculture (USDA) Agricultural Research Service (ARS) Sugarcane Research laboratory in Houma, Louisiana (Table 1). Larvae were reared individually on artificial diet at room temperature in the laboratory (25°C±2°C, 50% RH) until adult moths or parasitoids emerged [33]. Adult moths or parasitoids were then placed into individual vials and stored at −80°C for subsequent DNA studies. In eastern Texas, the collection site was at Beaumont, Texas within the Texas A&M Agrilife Research Center (Table 1). *Diatraea saccharalis* larvae from eastern Texas were field collected from *Saccharum* spp. (high fiber >20%, known as ‘energy cane’) throughout the growing season in 2009 and were similarly fed artificial diet until they became adults. In southern Texas, *D. saccharalis* larvae were rare on sugarcane plants. For this reason, live adult female *D. saccharalis* were used as lures to attract and trap adult males in July, August and September 2010 near a sugarcane mill in Santa Rosa, Texas (Table 1). Adult males were trapped on sticky cards, removed the following day, stored in 90% ethanol and were later frozen for DNA analyses. In Florida, adult male *D. saccharalis* moths were also collected using live adult females as lures. Florida samples were collected in August 2009 within the University of Florida Everglades Research and Education Center in Belle Glade, Florida (Table 1). Female *D. saccharalis* used as lures in Texas and Florida originated from a laboratory colony at the USDA ARS Sugarcane Research Laboratory unit in Houma, Louisiana. All adult moths were placed into 1.5 ml micro centrifuge tubes and stored at −80°C until used for DNA extraction.

**Table 1:** Collection localities for *D. saccharalis*.

Collection Location	Latitude/Longitude Coordinates	Number and Stage Collected	GenBank accession number or (BOLD) sequence ID number	Specimen name in Table 2, Figure 3
Santa Rosa S. Texas	26°15′24.26″N, 97°49′29.99″W	2 adult males	Males trapped with live female lures. GenBank accessions: KM288999, KM289000	US TxS 1 KM288999, US TxS 2 KM289000
Beaumont E. Texas	30°4′47.99″N, 94°17′40.34″W	2 adult males	Larvae collected on sugarcane, reared to adult. GenBank accessions: KM289001, KM289002	US TxE 1 KM289001, US TxE 2 KM289002
Burns Pt. Louisiana	29°43′46″N 91°26′32″W	1 adult male	Larvae collected on sugarcane, reared to adult. GenBank KM289003	US La1 KM289003
Ivanhoe Louisiana	29°47′35″N 91°42′24″W	1 adult male	Larvae collected on sugarcane, reared to adult. GenBank KM289004	US La2 KM289004
Belle Glade Florida	26°40′7.20″N, 80°37′57.63″W	2 adult males	Males trapped with live female lures. GenBank: KM289005, KM289006	US Fla1 KM289005, US Fla2 KM289006
Brazos Bend State Park Texas	29°22′51.6″N 95°35′43.43″W	3 adults	Bar Code of Life Database (BOLD#) BBLOC1560-11.COI-5P, BBLOC1565-11.COI-5P, BBLOD166-11.COI-5P	Tex166, Tex1560, Tex1565
Mexico	No coordinates	7 adults	GenBank accessions: JQ888360.1, JQ888359.1, JQ888358.1, JQ888357.1, JQ888356.1, JQ888355.1, JQ888354.1	Mex54–Mex60
Brazil	No coordinates	11 individuals (life stage not noted)	GenBank accessions: JN108986.1, JN108985.1, JN108984.1, JN108983.1, JN108982.1, JN108981.1, JN108980.1, JN108979.1, JN108978.1, JN108977.1, JN108976.1	Braz76–Braz86
Entre Rios Argentina	31°52′7.68″S 58°12′30.24″W	3 adults	Bar Code of Life Database MOTAR008-12.COI-5P, MOTAR077-12.COI-5P, MOTAR091-12.COI-5P	Arg008, Arg077, Arg091
Santa Cruz Bolivia	17°31′34.68″S 63 39′47.16″W	1 adult	Bar Code of Life Database IBLPY260-12.COI-5P	Boliv260
Santa Cruz Bolivia	17°29′56.76″S 63°39′9″W	1 adult	Bar Code of Life Database IBLPY275-12.COI-5P	Boliv275

Collections include individual *D. saccharalis* sequenced in this study from the southern United States, and accession numbers for COI sequences of *D. saccharalis* obtained from GenBank and BOLD. From our collections, the bar code was sequenced from 8 individuals (2 each from the four geographic regions in the southern United States-S. Texas, E. Texas, Louisiana, and Florida), and an additional 26 sequences were obtained from GenBank and BOLD.

### DNA extractions

Moths were identified as male or female *D. saccharalis* by examining genitalia. Only adult males were used for DNA comparisons. We used only one sex of moths (males) to ensure that any genetic differences we observed were not due to genetic differences which might exist between males and females. In addition, we used males rather than females since only males were attracted to the females in traps at the southern Texas and Florida sampling sites. The thorax of each male moth was removed and used for DNA extraction, while the abdomens were saved as vouchers and later used to prepare slides of the moth genitalia for species confirmation [1], [27], [34]. The Qiagen DNeasy Blood and Tissue kit (Valencia, California, USA) was used for DNA extraction, following the protocols suggested for animal tissue and using a 1 hour incubation with proteinase K at 65°C [35]. A Nanodrop 1000 spectrophotometer (Thermo Scientific, Pittsburgh, PA, USA) was used to measure the DNA concentration in ng/µl and purity (260/280 ratio). An Eppendorf Vacufuge was used to concentrate samples as needed in order to standardize the DNA concentration for all samples at 100±20 ng/µl prior to developing amplified fragment length polymorphisms (AFLPs).

### Amplified fragment length polymorphisms (AFLPs)

Amplified fragment length polymorphisms (AFLPs) were developed to compare the *D. saccharalis* collected from the regions described above in Texas, Louisiana, and Florida [36]. The specific protocol used is described in detail in Joyce et al. [37] and is condensed here. DNA from males collected from the four geographic regions was randomized on two 96-well plates for AFLP reactions. Each restriction/ligation reaction (well) consisted of the following: 0.05 µl each of EcoRI and MseI, 1.1 µl of T4 DNA ligase buffer, 1.1 µl of 0.5 M NaCl, 0.55 µl of diluted BSA (bovine serum albumin), 0.03 µl of T4 DNA ligase, 1.0 µl each of EcoRI and MseI adaptor pairs (Life Technologies, Carlsbad, Cal., USA), and 0.61 µl of sterile distilled water. Restriction/ligation reactions were held at room temperature overnight (ca. 12 h at 25°C) to ensure complete digestion [38]. The amplified product was diluted 20-fold using 15 mM Tris-HCl buffer (pH 8.0) containing 0.1 mM EDTA. Pre-selective PCR amplification was performed on an Applied Biosystems thermocycler (GeneAmp PCR System 9700). Each reaction contained 15 µl of AFLP Pre-selective Mix (Life Technologies, Carlsbad, Cal.), 1 µl of each amplification primer [i.e., EcoRI and MseI (Life Technologies, Carlsbad, Cal.)], along with 4 µl of the diluted restriction/ligation mixture. The PCR program for pre-selective amplification consisted of an initial warm-up of 95°C for 1 min followed by 20 cycles at 95°C for 20 s, 56°C for 30 s, and 72°C for 90 s with a final hold at 75°C for 5 min. The amplified product was diluted 20-fold using 15 mM Tris-HCl buffer (pH 8.0) containing 0.1 mM EDTA. Selective amplification was conducted using two primer combinations. For each selective amplification, a reaction consisted of 15 µl of AFLP Platinum Supermix, 1.0 µl of EcoRI selective primer, and 1.0 µl of MseI selective primer. Two selective primer combinations were used (1) M-CAT/E-ACT, and (2) M-CAC/E-ACG (Life Technologies, Carlsbad, Cal.). The PCR program for selective amplification consisted of an initial warm-up of 95°C for 1 min, 12 cycles of 95°C for 20 s, 65°C for 40 s with a lowering of 0.7°C per cycle, 72°C for 90 s, followed by 35 cycles of 95°C for 20 s, 56°C for 40 s, 72°C for 90 s, and a final hold of 72°C for 7 min before storing the samples at 4°C. Prior to capillary electrophoresis, 9 µl of HiDi® formamide and 0.5 µl of the Genescan 400HD ROX size standard (Life Technologies, Carlsbad, Cal.) were added to 1 µl of the final product of each sample. Sample fragments were separated using automated capillary electrophoresis by an ABI 3100 automated capillary DNA sequencer.

GeneMapper version 4.0 (Life Technologies, Carlsbad, Cal.) was used to determine presence or absence of fragments. Peaks were examined by eye to ensure the peak detection threshold was at least 1.5 times higher than the mean background level. The peak detection threshold was set for each primer combination, and was typically 100 luminescent units. Each AFLP marker was considered a locus and assumed to have two possible alleles (0 = absent, 1 = present). Bands not present in more than one individual were eliminated (i.e., private alleles) prior to further analyses, as they were not considered informative. The SESim method [39] was used to determine the number of individuals and markers needed in order to adequately represent the genetic variation of the populations sampled in this study. A SESim value <0.05 indicates consistency in the clustering pattern produced by a specific combination of markers and individuals for the studied organism at the geographic scale considered [39]. Structure 2.2 software [40] was used to group individuals with similar genotypes within each species. Structure 2.2 uses a Bayesian algorithm to cluster individuals into K, which is defined as the number of genetically distinct populations in a data set. Parameters used for this analysis include the following: no a priori assignment of individuals to a known population, analysis for diploid insects, a burn-in of 10 000 iterations, an admixture model, and independent loci. The number of potential populations for K was estimated as the number of geographic sampling locations (4) plus 4 (K = 8) as suggested by Pritchard et al. [41], and each iteration was run 20 times. At the completion of Structure 2.2 runs, ΔK was calculated for each species using the method of Evanno et al. [42], to determine the most likely number of population clusters (K) for each species.

### Mitochondrial DNA cytochrome oxidase I (COI)

A 658 base pair region (the ‘bar code’) of the mitochondrial COI gene region was sequenced from two individuals from each of the four geographic regions sampled in this study from the southern US. The purpose was to compare the COI sequences of our samples with those available for *D. saccharalis* available in GenBank and the Bar Code of Life Data System (BOLD) databases [43], to determine if our sampled populations in the southern US may be genetically similar to any individual *D. saccharalis* included in those databases, and to determine the number of genetically divergent lineages for the sequences available for *D. saccharalis*. The DNA used for sequencing COI was extracted from male *D. saccharalis* as described above using the Qiagen DNeasy Blood and Tissue kit (*see DNA extraction*).

The barcode region of the COI gene was amplified using primers for the mitochondrial DNA ‘bar code’ of Lepidoptera described in Hajibabaei et al. [44]. The sequence of the forward primer LepF was 5_-ATTCAACCAATCATAAAGATATTGG-3 and the reverse primer sequence of LepR was 5_-TAAACTTCTGGATGTCCAAAAAATCA-3 (Life Technologies, Carlsbad, Cal.). The touchdown PCR program consisted of an initial 2 minutes at 95°C, then 12 cycles of 95°C for 10 sec, 58–46°C for 10 sec with a lowering of 1°C temperature each cycle, and 72°C for 60 seconds. Following PCR, samples were cleaned up using a USB Exo-sap-it (Affymetrix, Inc., Santa Clara, Cal.) PCR cleanup kit. Sequencing was carried out using the Big Dye Terminator v3.1 Cycle Sequencing Kit (Life Technologies, Carlsbad, Cal.) followed by fractionation on an ABI 3730XL Genetic Analyzer.

DNA sequences were edited using Geneious 7.0 (Biomatters, Aukland, New Zealand). The forward and reverse sequences for each individual were assembled into a consensus sequence. We aligned our consensus sequences from *D. saccharalis* collected in the southern United States with 26 other *D. saccharalis* obtained from GenBank and BOLD (Table 1). Alignments were made in Geneious 7.0 using the Clustal W alignment function, and Tamura-Nei genetic distances were calculated and used to produce a neighbor joining tree using midpoint rooting. Bootstrap support values were obtained by 500 pseudoreplicates of the aligned dataset.

## Results

### Morphological identification of *D. saccharalis*

**Figure 1:**
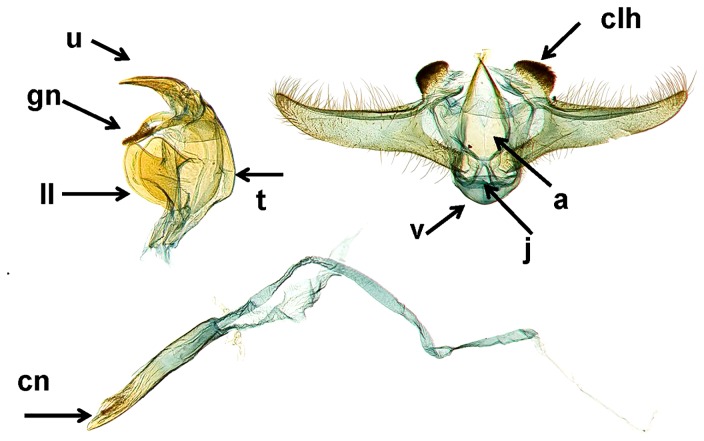
Male genitalia of *Diatraea saccharalis*. Adult male collected in Avoyelles, Louisiana from sugarcane by R T Richard. Adult is deposited in the National Museum of Natural History, Smithsonian Institute, Washington DC (USNM Slide #112, 735). Terminology is from Dyar and Heinrich (1927), an = anellus, chl = basal projection lobe from costa of harpe, cn = cornatus (or cornuti) of penis, gn = gnathos, j = juxta, ll = lateral lobe of tegumen, t = tegumen, u = uncus, v = vinculum. Photo edited by M Metz and M A Solis.

All the adult male moths from the four geographic areas sampled (southern Texas, eastern Texas, Louisiana and Florida) were identified to *Diatraea saccharalis*, based on the morphology of the male genitalia [1](Figure 1).

### AFLPS

**Figure 2:**
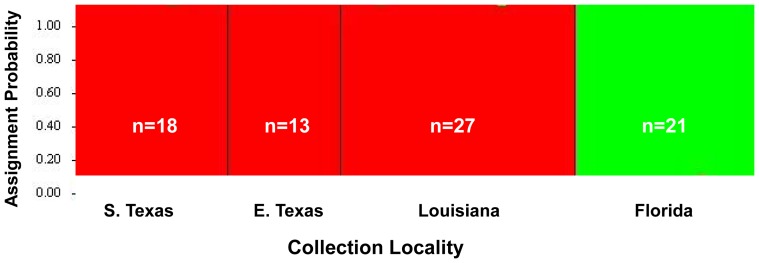
Structure 2.2 analysis depicts two genetically distinct clusters of *D. saccharalis*. Individuals from southern Texas (S. Texas), eastern Texas (E. Texas) and Louisiana grouped together within the red cluster, while individuals from Florida grouped within the green cluster. The y-axis shows the probability of each individual to belong to a genetically distinct cluster. The number of individuals from each region used for the analysis is represented by ‘n’.

A total of 79 *D. saccharalis* male adults (18 from southern Texas, 13 from eastern Texas, 27 from Louisiana, and 21 from Florida) and two primer combinations (M-CAT/E-ACT; M-CAC/E-ACG) were used to obtain 96 AFLP markers. This number of individuals and markers were found to be sufficient in order to adequately represent population genetic structure of this insect in the sampled regions [39]. Structure 2.2 analyses clearly depict two genetically distinct clusters of *D. saccharalis* present in the southern United States (Figure 2). The presence of two distinct clusters was confirmed using the ΔK statistic of Evanno et al. [42]. *Diatraea saccharalis* from southern Texas, eastern Texas and Louisiana grouped together, whereas individuals from Florida belong to a genetically distinct cluster. Our data show no evidence of interbreeding or migration between the two genetic clusters, suggesting that the Florida population of *D. saccharalis* is a distinct genotype and possibly a cryptic species. Of the 96 alleles produced by the AFLP reactions, 24 were present only in Texas and Louisiana, while 14 alleles were unique to the Florida population.

### Mitochondrial DNA COI barcode sequences

**Figure 3:**
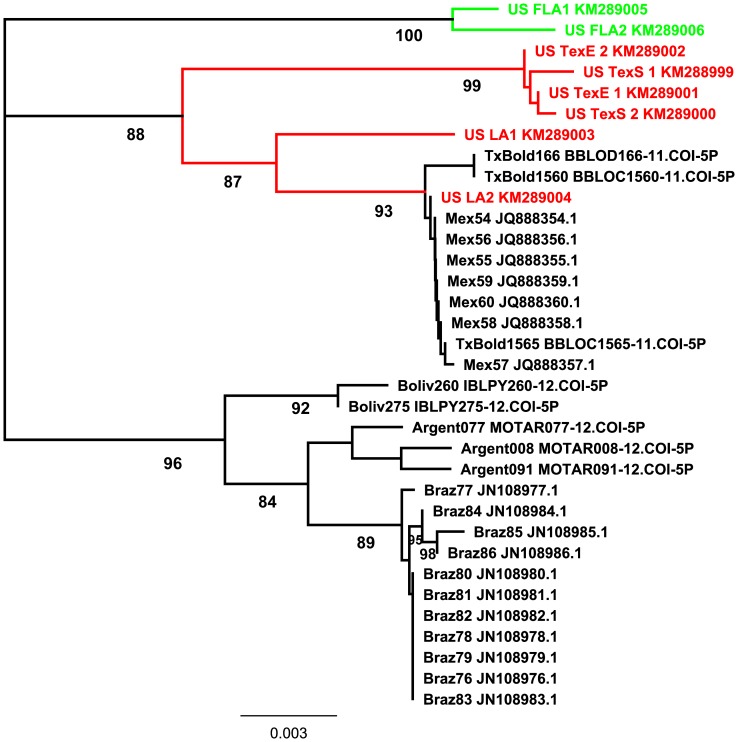
Neighbor joining phylogram of *D. saccharalis* populations. The phylogram is based on a neighbor joining analysis of 658 bp of the COI barcode region. Bootstrap support values are based on 500 pseudoreplicates, and those above 80% are shown below supported nodes. Individuals collected as part of this study are shown in color: Florida in green; Texas/Louisiana in red. Individuals shown in black were obtained from GenBank and BOLD databases. See Table 1 for specimen details.

A neighbor joining tree based on the 658 base pair barcoding region of the COI mitochondrial gene shows the presence of three genetically distinct clusters of *D. saccharalis* in the Western Hemisphere (Figure 3). The neighbor joining tree was generated using the COI sequences of two individuals of *D. saccharalis* from each of the four geographic regions sampled in the southern United States in this study and also using *D. saccharalis* sequences obtained from GenBank and BOLD. Sequences from these databases represent *D. saccharalis* from throughout the Western Hemisphere and were included to produce a more informative tree (Table 1). *Diatraea saccharalis* from Florida grouped together within a distinct cluster. A second cluster consists of individuals from Texas and Louisiana collected in this study, as well as *D. saccharalis* from Texas and Mexico obtained from GenBank and BOLD. The third cluster in the neighbor joining tree consists of *D. saccharalis* from South America, specifically from Bolivia, Argentina and Brazil, obtained from GenBank and BOLD (Table 1, Figure 3).

The genetically divergent cluster of *D. saccharalis* from Florida is supported by both the AFLP data (Figure 2) and the COI data (Figure 3). Pairwise genetic distances of *D. saccharalis* COI sequences from Florida compared to Texas and Louisiana ranged from 2.8 to 3.4% (Table 2). Similarly, pairwise genetic distances between Florida and Mexico and between Florida and South America ranged between 2.7 and 3.0%. In contrast, pairwise genetic distances between southern and eastern Texas individuals were low (0–2%). Individuals from Texas and Louisiana show moderate genetic distances (1.5–2.3%) as do those between Mexico and Texas (1.7 to 1.8%). Texas populations clustered together with *D. saccharalis* from Louisiana in the AFLP analysis, demonstrating interbreeding and gene flow with those populations. *Diatraea saccharalis* from South America grouped within a separate cluster. Genetic distances among South American samples ranged from 1–1.2%, while pairwise genetic distances between South American and North American *D. saccharalis* ranged from 2.5 to 3.2%.

**Table 2:** Genetic distance between *D. saccharalis* populations.

	Arg008	Arg077	Arg091	Boliv260	Boliv275	Braz76	Braz77	Braz78	Braz79	Braz80	Braz81	Braz82	Braz83	Braz84	Braz85	Braz86	USTxS1
**Arg008**		0.005	0.003	0.012	0.011	0.008	0.008	0.008	0.008	0.008	0.008	0.008	0.008	0.008	0.008	0.008	0.031
**Arg077**	0.005		0.005	0.011	0.009	0.006	0.006	0.006	0.006	0.006	0.006	0.006	0.006	0.006	0.006	0.006	0.030
**Arg091**	0.003	0.005		0.012	0.011	0.008	0.008	0.008	0.008	0.008	0.008	0.008	0.008	0.008	0.008	0.008	0.031
**Boliv260**	0.012	0.011	0.012		0.002	0.011	0.011	0.011	0.011	0.011	0.011	0.011	0.011	0.011	0.011	0.011	0.031
**Boliv275**	0.011	0.009	0.011	0.002		0.009	0.009	0.009	0.009	0.009	0.009	0.009	0.009	0.009	0.009	0.009	0.030
**Braz76**	0.008	0.006	0.008	0.011	0.009		0.001	0.000	0.000	0.000	0.000	0.000	0.000	0.000	0.002	0.000	0.030
**Braz77**	0.008	0.006	0.008	0.011	0.009	0.001		0.001	0.001	0.001	0.001	0.001	0.001	0.001	0.003	0.001	0.030
**Braz78**	0.008	0.006	0.008	0.011	0.009	0.000	0.001		0.000	0.000	0.000	0.000	0.000	0.000	0.002	0.000	0.030
**Braz79**	0.008	0.006	0.008	0.011	0.009	0.000	0.001	0.000		0.000	0.000	0.000	0.000	0.000	0.002	0.000	0.030
**Braz80**	0.008	0.006	0.008	0.011	0.009	0.000	0.001	0.000	0.000		0.000	0.000	0.000	0.000	0.002	0.000	0.030
**Braz81**	0.008	0.006	0.008	0.011	0.009	0.000	0.001	0.000	0.000	0.000		0.000	0.000	0.000	0.002	0.000	0.030
**Braz82**	0.008	0.006	0.008	0.011	0.009	0.000	0.001	0.000	0.000	0.000	0.000		0.000	0.000	0.002	0.000	0.030
**Braz83**	0.008	0.006	0.008	0.011	0.009	0.000	0.001	0.000	0.000	0.000	0.000	0.000		0.000	0.002	0.000	0.030
**Braz84**	0.008	0.006	0.008	0.011	0.009	0.000	0.001	0.000	0.000	0.000	0.000	0.000	0.000		0.000	0.000	0.030
**Braz85**	0.008	0.006	0.008	0.011	0.009	0.002	0.003	0.002	0.002	0.002	0.002	0.002	0.002	0.000		0.000	0.031
**Braz86**	0.008	0.006	0.008	0.011	0.009	0.000	0.001	0.000	0.000	0.000	0.000	0.000	0.000	0.000	0.000		0.030
**USTxS1**	0.031	0.030	0.031	0.031	0.030	0.030	0.030	0.030	0.030	0.030	0.030	0.030	0.030	0.030	0.031	0.030	
**USTxS2**	0.030	0.028	0.030	0.030	0.028	0.029	0.029	0.029	0.029	0.029	0.029	0.029	0.029	0.027	0.029	0.028	0.002
**USTxE1**	0.030	0.028	0.030	0.030	0.028	0.028	0.028	0.028	0.028	0.028	0.028	0.028	0.028	0.028	0.030	0.029	0.001
**USTxE2**	0.030	0.028	0.030	0.030	0.028	0.028	0.028	0.028	0.028	0.028	0.028	0.028	0.028	0.028	0.028	0.029	0.001
**USLa1**	0.025	0.027	0.022	0.028	0.027	0.027	0.027	0.027	0.027	0.027	0.027	0.027	0.027	0.027	0.027	0.027	0.018
**USLa2**	0.024	0.026	0.024	0.026	0.024	0.026	0.026	0.026	0.026	0.026	0.026	0.026	0.026	0.026	0.026	0.026	0.017
**USFla1**	0.030	0.028	0.030	0.027	0.025	0.028	0.028	0.028	0.028	0.028	0.028	0.028	0.028	0.028	0.028	0.029	0.033
**USFla2**	0.031	0.030	0.031	0.025	0.027	0.029	0.029	0.029	0.029	0.029	0.029	0.029	0.029	0.030	0.033	0.030	0.034
**Mex55**	0.025	0.027	0.025	0.025	0.023	0.026	0.026	0.026	0.026	0.026	0.026	0.026	0.026	0.026	0.026	0.027	0.018
**Mex56**	0.025	0.027	0.025	0.025	0.023	0.026	0.026	0.026	0.026	0.026	0.026	0.026	0.026	0.026	0.026	0.027	0.018
**Mex57**	0.025	0.027	0.025	0.025	0.024	0.028	0.028	0.028	0.028	0.028	0.028	0.028	0.028	0.027	0.027	0.027	0.020
**Mex58**	0.025	0.027	0.025	0.025	0.024	0.027	0.027	0.027	0.027	0.027	0.027	0.027	0.027	0.027	0.027	0.027	0.019
**Mex59**	0.025	0.027	0.025	0.025	0.024	0.027	0.027	0.027	0.027	0.027	0.027	0.027	0.027	0.027	0.027	0.027	0.019
**Mex60**	0.025	0.027	0.025	0.025	0.024	0.027	0.027	0.027	0.027	0.027	0.027	0.027	0.027	0.027	0.027	0.027	0.019
**Mex54**	0.025	0.027	0.025	0.025	0.023	0.026	0.026	0.026	0.026	0.026	0.026	0.026	0.026	0.026	0.026	0.027	0.018
**Tx166**	0.023	0.025	0.023	0.027	0.025	0.029	0.029	0.029	0.029	0.029	0.029	0.029	0.029	0.029	0.029	0.029	0.002
**Tx1560**	0.023	0.025	0.023	0.027	0.025	0.029	0.029	0.029	0.029	0.029	0.029	0.029	0.029	0.029	0.029	0.029	0.002
**Tx1565**	0.025	0.027	0.025	0.025	0.023	0.027	0.027	0.027	0.027	0.027	0.027	0.027	0.027	0.027	0.027	0.027	0.019

	USTxS2	USTxE1	USTxE2	USLa1	USLa2	USFla1	USFla2	Mex55	Mex56	Mex57	Mex58	Mex59	Mex60	Mex54	Tx166	Tx1560	Tx1565
**Arg008**																	
**Arg077**																	
**Arg091**																	
**Boliv260**																	
**Boliv275**																	
**Braz76**																	
**Braz77**																	
**Braz78**																	
**Braz79**																	
**Braz80**																	
**Braz81**																	
**Braz82**																	
**Braz83**																	
**Braz84**																	
**Braz85**																	
**Braz86**																	
**USTxS1**																	
**USTxS2**	**-**																
**USTxE1**	0.000	**-**															
**USTxE2**	0.000	0.000	**-**														
**USLa1**	0.017	0.017	0.017	**-**													
**USLa2**	0.015	0.015	0.015	0.009	**-**												
**USFla1**	0.031	0.031	0.031	0.029	0.028	**-**											
**USFla2**	0.034	0.032	0.032	0.031	0.030	0.005	**-**										
**Mex55**	0.017	0.017	0.017	0.009	0.000	0.026	0.027	**-**									
**Mex56**	0.017	0.017	0.017	0.009	0.000	0.026	0.027	0.000	**-**								
**Mex57**	0.019	0.019	0.017	0.009	0.000	0.027	0.030	0.000	0.000	**-**							
**Mex58**	0.017	0.017	0.017	0.009	0.000	0.027	0.028	0.000	0.000	0.000	**-**						
**Mex59**	0.017	0.017	0.017	0.009	0.000	0.027	0.028	0.000	0.000	0.000	0.000	**-**					
**Mex60**	0.017	0.017	0.017	0.009	0.000	0.027	0.028	0.000	0.000	0.000	0.000	0.000	**-**				
**Mex54**	0.017	0.017	0.017	0.009	0.000	0.026	0.027	0.000	0.000	0.000	0.000	0.000	0.000	**-**			
**Tx166**	0.019	0.019	0.019	0.011	0.002	0.028	0.030	0.002	0.002	0.002	0.002	0.002	0.002	0.002	**-**		
**Tx1560**	0.019	0.019	0.019	0.011	0.002	0.028	0.030	0.002	0.002	0.002	0.002	0.002	0.002	0.002	0.000	**-**	
**Tx1565**	0.017	0.017	0.017	0.009	0.000	0.027	0.028	0.000	0.000	0.000	0.000	0.000	0.000	0.000	0.002	0.002	**-**

Distance values were produced using Geneious 7.0 software. Differences are reflected in the neighbor joining tree in Figure 3. Arg = Argentina Boliv = Bolivia, Braz = Brazil, Mex = Mexico, Tx = TexasBOLD, USFla = Florida, United States, USLa = Louisiana, United States. USTxE = East Texas, United States, USTxS = South Texas.

## Discussion and Conclusions

*Diatraea saccharalis* has been considered one species in the southern US and throughout the Western Hemisphere for several centuries. This moth is nocturnal, has few distinctive visual markings, and is geographically widespread. Insects with these characteristics are prone to be part of cryptic species complexes [45]. The adults collected in this study were all identified to *D. saccharalis* based on the morphology of the adult male genitalia [1]. However, significant genetic divergence between lineages suggests the presence of a cryptic species complex.

We used two molecular markers (AFLP and COI sequences) to examine variation in the population structure of *D. saccharalis* in the southern United States. Both AFLP and COI markers characterized a genetically distinct cluster of *D. saccharalis* from Florida. Mitochondrial DNA generated genetic distances between Florida and other *D. saccharalis* populations in the range of 2.5–3%. This degree of genetic divergence suggests that Florida *D. saccharalis* could represent a distinct species [30], [46]. The Florida *D. saccharalis* population could belong to a lineage that includes Caribbean populations from the Greater Antilles, such as Cuba, Puerto Rico, Hispaniola, and Jamaica, islands which are thought to be of a relatively similar geologic age [47]. Based on the data from this study and other public sequences, the Florida population does not appear to have been introduced into the southern United States from Mexico or South America. Comparisons between Florida and Caribbean populations would shed light on the origin of Florida *D. saccharalis* populations. The Louisiana and Texas populations of *D. saccharalis* group together in the same cluster as those from Mexico, suggesting they may have been introduced from Mexico perhaps through other introductions of sugarcane host plant material or within storms cells. Avequin [11] suggested *D. saccharalis* in Louisiana originated from the introduction of sugarcane in Louisiana from Hispaniola (Haiti/Dominican Republic) in 1751. However, *D. saccharalis* was not recorded as a pest in Louisiana until 1855. In the early 1800s, additional sugarcane varieties were introduced into Louisiana [11], and these could have been a source for the *D. saccharalis* introduced into Louisiana.

The mitochondrial COI data provide evidence for at least three distinct lineages in the Western Hemisphere: A Florida lineage, a lineage including Texas, Louisiana and Mexico, and a third lineage from South America that includes Brazil, Argentina, and Bolivia. A fourth divergent group of *D. saccharalis* in Colombia is suggested by Palacio-Cortes et al. [29]. Finally, populations of *D. saccharalis* from the Caribbean could comprise an additional lineage or could group together with the Florida cluster. Genetic distances among the three lineages of *D. saccharalis* depicted in Fig. 3 fall within a range of 0.025–0.03, nearly ten times higher than the genetic distance values observed within any one of the three lineages, a level of difference which suggests the lineages are distinct species [30], [48]. Previous work by Pashley et al. [21] found that populations of *D. saccharalis* from Louisiana and Texas are genetically divergent from those in Brazil [21], and likely consist of two distinct species. Our study, the work of Pashley et al. [21], and sequences from South America in GenBank and BOLD all support the existence of at least three divergent lineages.

In the last decade, DNA barcoding has provided a method to assess genetic diversity within and among species. Intraspecific genetic diversity of mitochondrial COI in *Plutella xylostella* (L.), the diamond back moth, averaged ∼1%, which fell within the range of expected intraspecific variation [49]. In contrast, interspecific variation in COI sequences among *Choristoneura* (Lepidoptera: Tortricidae) species ranged from 1–2% [46]. In several cases, insects with broad geographic distributions have been found to belong to cryptic species complexes [22], [30], [50], [51], [52], [53]. In the case of the butterfly *Astraptes fulgerator* (Walch)(Lepidoptera: Hesperiidae) [30], genetically divergent lineages parallel observed variation in larval coloration and host plant preferences. In this species complex, interspecific genetic divergence among ten taxa was ∼2.97%, while within species genetic divergence was typically less than 1% [30]. Adult *A. fulgerator* from all the studied populations had identical genitalia and adults provided little indication of divergent lineages until genetic variation was explored within the group. The 2–3% divergence we have found among the *D. saccharalis* lineages we have identified suggests they are distinct species. The three lineages we have identified are geographically structured (i.e., Florida; Texas/Louisiana/Mexico; South America). To be robust, defining species limits should include multiple lines of evidence. Such an approach is referred to as integrative taxonomy [54], [55] and should include morphological, behavioral, molecular and geographic data [56]. Thus, although our data strongly suggests the existence of a *D. saccharalis* cryptic species complex, further lines of evidence would provide additional support of this assertion.

We originally suspected the existence of more than one genotype of *D. saccharalis* in the southern US due to the differential success of natural enemies which had been introduced into the region. We suspected Louisiana *D. saccharalis* populations would be divergent from Texas and/or Florida populations. However, our data show that Louisiana and Texas belong to the same genetic cluster while Florida constitutes a divergent genotype. Our data suggest that the difference in establishment of parasitoids of *D. saccharalis* in Texas or Louisiana is unlikely to be due to difference in *D. saccharalis* genotypes, but could be influenced by climatic differences or cultural practices which vary through the southern US. For example, in Louisiana, sugarcane fields are harvested in the fall, leaving little vegetation for parasitoids to overwinter, which could reduce parasitoid establishment. In contrast, sugarcane is grown year round in Texas and Florida [31].

We used live female *D. saccharalis* from Louisiana as a lure to attract and trap Florida *D. saccharalis* male moths, yet we found that Louisiana moths are genetically divergent from the Florida moths. Moths from Louisiana and Florida are genetically distinct, yet the pheromones from Louisiana females were effective at attracting Florida males. Although we observed genetic diversity in *D. saccharalis* collections between the two genetically distinct clusters, trapping adults does not allow us to associate the individuals we collected with particular host plants. In order to determine if host plant associated strains exist for *D. saccharalis*, one would need to collect larvae from multiple host plants and examine the genetic differences among the host plant associated populations. Pheromones can cross-attract between species, especially if populations evolved in allopatry, where there is no selective pressure for signal divergence [57], [58].The pheromone blends of Brazilian *D. saccharalis* have been investigated, and variation exists among populations; unfortunately, relative attraction of these blends to different *D. saccharalis* populations has not yet been tested [29], [59].

The potential cryptic lineages of *D. saccharalis* we have identified in this study deserve further attention. This insect is considered a major pest throughout the Western Hemisphere and has been easily confused with other species of *Diatraea* based on morphology. Genetically distinct lineages may differ in their damage potential and/or in their vulnerability to pest control strategies such as biological control. The ability to characterize and identify genotypes of *D. saccharalis* and related species or as of yet undiscovered species will improve pest management efforts against this pest and improve area-wide control efforts across its geographic distribution. Additional research on the population genetics of *D. saccharalis* in Central America and the Caribbean will further our understanding of its geographic population structure and clarify the composition of this potential cryptic species complex.
